# Modeling the Dynamics of a Non-Limited and a Self-Limited Gene Drive System in Structured *Aedes aegypti* Populations

**DOI:** 10.1371/journal.pone.0083354

**Published:** 2013-12-10

**Authors:** Mathieu Legros, Chonggang Xu, Amy Morrison, Thomas W. Scott, Alun L. Lloyd, Fred Gould

**Affiliations:** 1 Department of Entomology, North Carolina State University, Raleigh, North Carolina, United States of America; 2 Department of Entomology, University of California Davis, Davis, California, United States of America; 3 Institut für Integrative Biologie, ETH Zürich, Zürich, Switzerland; 4 Division of Earth and Environmental Sciences, Los Alamos National Laboratory, Los Alamos, New Mexico, United States of America; 5 Fogarty International Center, National Institutes of Health, Bethesda, Maryland, United States of America; 6 Department of Mathematics and Biomathematics Graduate Program, North Carolina State University, Raleigh, North Carolina, United States of America; Virginia Tech, United States of America

## Abstract

Recently there have been significant advances in research on genetic strategies to control populations of disease-vectoring insects. Some of these strategies use the gene drive properties of selfish genetic elements to spread physically linked anti-pathogen genes into local vector populations. Because of the potential of these selfish elements to spread through populations, control approaches based on these strategies must be carefully evaluated to ensure a balance between the desirable spread of the refractoriness-conferring genetic cargo and the avoidance of potentially unwanted outcomes such as spread to non-target populations. There is also a need to develop better estimates of the economics of such releases. We present here an evaluation of two such strategies using a biologically realistic mathematical model that simulates the resident *Aedes aegypti* mosquito population of Iquitos, Peru. One strategy uses the selfish element *Medea*, a non-limited element that could permanently spread over a large geographic area; the other strategy relies on Killer-Rescue genetic constructs, and has been predicted to have limited spatial and temporal spread. We simulate various operational approaches for deploying these genetic strategies, and quantify the optimal number of released transgenic mosquitoes needed to achieve definitive spread of *Medea*-linked genes and/or high frequencies of Killer-Rescue-associated elements. We show that for both strategies the most efficient approach for achieving spread of anti-pathogen genes within three years is generally to release adults of both sexes in multiple releases over time. Even though females in these releases should not transmit disease, there could be public concern over such releases, making the less efficient male-only release more practical. This study provides guidelines for operational approaches to population replacement genetic strategies, as well as illustrates the use of detailed spatial models to assist in safe and efficient implementation of such novel genetic strategies.

## Introduction

The recent resurgence of dengue as a serious public health issue in many countries around the world [1–3] has reinforced the need for efficient and sustainable methods of vector control, particularly since there is currently no practical vaccine or prophylactic drug available against dengue viruses. *Aedes aegypti*, the main dengue-vectoring mosquito, is therefore the target of important vector control efforts. Pest control programs directed against this species have been ongoing for several decades, but with limited success [4,5]. Substantial research has been aimed at improving control strategies, and in the wake of recent major breakthroughs in genetic engineering, several promising strategies are being explored that use genetically modified mosquitoes (GMMs) to control dengue transmission [6–8]. 

 In some situations GMM strains could be used as population suppression tools [9,10]. This engineering approach is conceptually similar to but potentially more efficient than the classical Sterile Insect Technique (SIT) or classical female-killing methods [11]. In the current study we focus on the use of GMMs as population replacement tools [8] where a resident, disease-vectoring population of mosquitoes is replaced by engineered mosquitoes that do not transmit dengue virus. *Aedes aegypti* lines that are resistant to infection by one dengue virus serotype have been successfully engineered using RNA interference [12,13], although in one case the resistance phenotype was lost after 17 generations [14]. It is likely that these lab-created, genetically engineered resistant strains will suffer from a somewhat reduced fitness compared to their wild-type counterpart and could not, on their own, increase from low to high frequency within wild populations. In order to achieve population replacement without repeated GMM releases, these virus resistance-conferring genetic inserts must be linked to a selfish transgenic element that inherently increases its frequency in a population without conferring higher fitness to organisms that contain it [8]. This process is often referred to as genetically engineered gene drive. 

 A number of genetic mechanisms can confer gene driving properties [8]. Some are known to occur naturally and can be re-engineered as drivers for closely linked anti-pathogen genes [15–21], while other mechanisms have been conceived to be built specifically for this purpose [22–24]. We chose to focus our study on two specific gene drive systems that are expected to differ substantially in their dynamics: Medea (Maternal Effect Dominant Embryonic Arrest), a selfish genetic mechanism first discovered in *Tribolium* *spp.* [15], and Killer-Rescue (KR), an artificial system for self-limited drive of desirable genes [24]. Both a *Medea* element and a KR system have been successfully constructed and tested in laboratory populations of *Drosophila* [25,26]. 

 The mechanism of gene drive in *Medea* elements requires a single DNA construct that includes both a coding sequence for an embryo-specific toxic substance that is expressed in the female germline and a coding sequence for an antidote to the toxin that is transcribed early during embryogenesis [25]. A female that is heterozygous for the *Medea* element only passes the construct on to half of her embryos but transmits the toxin to all offspring. The embryos that do not inherit the *Medea* element from their mother or father cannot produce the antidote for the maternally transmitted toxin, and they die. This differential mortality of individuals without the *Medea* element increases the *Medea* frequency in the population [27]. If the construct with the *Medea* element also contains an anti-pathogen gene, its frequency will also increase. Simple, deterministic models predict that a *Medea* element without a fitness cost (beyond embryo lethality) will become fixed in a large population even when released at very low frequencies [27,28]. If there are additional fitness costs, wildtype individuals in a population can still be completely eliminated if there is one release of a larger number of *Medea*-bearing individuals. It can be inferred from these models that a single point release of *Medea* could result in geographical spread to all populations that are connected by any gene flow.

 Spreading of a *Medea* element and the linked anti-pathogen gene throughout the range of a pathogen-vectoring mosquito could be seen as a positive characteristic as long as there were no potential environmental, political, or health risks associated with this spread. Politically, unless all potentially affected countries agreed to release of a transgenic mosquito with a *Medea* element, no single country could legitimately carry out a release. Furthermore, even if an anti-pathogen gene were successfully driven into a population by a genetic strategy like *Medea*, it would be required to be certifiably genetically and evolutionarily stable. If the disease resistance were to fail after introgression in the population, the loss of herd immunity resulting from the temporary protection could cause reoccurring disease outbreaks to be much more severe. While this is a general concern for any successful disease control strategy (other than those specifically designed to build immunity like vaccination), the genetic nature of the approaches considered here make such a failure potentially more likely, as demonstrated by the unexpected failure of one anti-dengue construct after 17 generations [14], 

 A potential approach to alleviate some of these concerns would rely on the use, at least as a first step, of self-limiting genetic strategies, i.e. approaches designed to spread desirable genetic cargo in a fashion that is limited in space and time. Spatial limitations could restrict spread to delimited test area, while temporal limitations would permit short-term studies (notably as proof of principle) where concerns about potential long-term effects are not relevant. 

This is the framework in which the KR gene drive system was developed. This mechanism requires one genetic construct that codes for a “Killer” toxin in any or all tissues and times, and a second, genetically unlinked construct that codes for production of a “Rescue” antidote in at least all of the tissues and times that the toxin is present. The “Rescue” allele (R) confers therefore a strong advantage in a genetic background that includes a “Killer” allele (K), whereas no advantage is provided if the K allele is absent. On the other hand, the K allele is by definition strongly selected against (from its autocidal effect), unless in a genetic background that includes an R allele. At the population level, this means that K and R alleles are subject to frequency-dependent selection, in a complex fashion that depends on the frequencies of both alleles in the population (and on their linkage disequilibrium). 

The dynamics of this system have been studied in detail by a deterministic mathematical model [24] leading to two main predictions. First, this system can theoretically spread an anti-pathogen gene into the population if it is linked to the R allele. High frequencies (required for significant protection against disease) can be achieved with multiple large releases, and continued releases are necessary for sustaining such high frequencies. Second, as long as there is any fitness cost associated with the R construct or the genetically linked anti-pathogen gene, the spread will be spatially limited and all of the engineered constructs will eventually be lost from the population. Even if there were no costs to the transgenes, the KR system would be spatially limited in its spread. This KR gene drive system could therefore be used to test the efficacy of an anti-pathogen gene in a realistic field environment, but could be designed to be temporally and spatially limited (in particular by designing a system where the R allele is associated with a fitness cost). 

 Analyses of simple deterministic models of *Medea* and KR have been useful for exploring the impacts of factors such as fitness costs, sibling competition, dominance of killing and rescue mechanisms, and sex-specificity of killing. However, the thorough regulatory assessments conducted before environmental release of any engineered mosquitoes will likely require analysis with more detailed, biologically realistic models. We previously described a spatially explicit, stochastic model of the population dynamics and genetics of *Ae. aegypti* [29] and compared its predictions to the actual population dynamics of this mosquito in the tropical city of Iquitos, Peru [30].

 We have used this model, Skeeter Buster, to examine the potential of a transgenic female-killing *Ae. aegypti* strain for suppressing or eradicating local mosquito populations [31]. Predictions of this model contrast substantially with those of simple models in part because of the heterogeneity among houses in the city, very restricted movement of this mosquito species, and because only a fraction of the resident mosquitoes are available for mating at any point in time. 

 In the current study, we use Skeeter Buster to examine operational factors in transgenic release programs that cannot be evaluated with simpler *Medea* and KR gene drive models. We simulate a number of possible release strategies for the transgenic mosquitoes in terms of life stage released, sex released, number of releases over time, and spatial distribution of release locations. 

## Materials and Methods

### Model description and study area

A detailed description of the Skeeter Buster model has been published [29]. The model can be downloaded directly from http://www.skeeterbuster.net and the source code is available on demand from the authors via this same website. In this section we only provide a summary of the main features of the model. Skeeter Buster is a stochastic model that simulates cohorts of immature mosquitoes in individual water-holding containers, and simulates individual adults in houses. The development of cohorts at each life stage (eggs, larvae, pupae) is described by a temperature-dependent enzyme kinetics model [32] and the intraspecific competition at larval stages is implemented by tracking the amount of nutritional resources in each container, which in turn affects larval growth, survival rates and adult size [33]. Skeeter Buster is a spatial model, where houses are laid out on a rectangular grid. Dispersal is limited to nearest neighbor houses on each day, with a daily dispersal probability of 30% for each adult as found in a previous calibration study [29]. Dispersal direction is chosen at random. Nearest neighbors are the orthogonally-adjacent houses on the grid (von Neumann neighborhood), and houses on the edges have no neighbor beyond the edge. Adult females are assumed to be strictly monogamous [34]. Unmated females mate on the first day that they are located in the same house as one or more adult males. If several males are present in the same house, a mate is chosen with a probability proportional to its size. 

 For the purpose of evaluating the spread of gene drive elements in a specific natural mosquito population, we chose to simulate the situation of the city of Iquitos, Peru. The distribution of houses in Iquitos is similar to the grid in the model, and the data on *Ae. aegypti* population dynamics in the city are extensive [30,35,36]. In a previous study, we have described the calibration of Skeeter Buster to the Iquitos case and the ability of the model to reliably simulate this mosquito population [30]. In this study we use the model calibrated in a similar fashion. We simulate a 612-house area within the city, located in the Maynas neighborhood where mosquito population levels and dengue incidence are among the highest in the city. Container distribution in the simulated area is taken directly from data collected in 153 houses (replicated randomly 4 times in the simulations) during entomological surveys carried out in the city [30]. Weather conditions are obtained from NOAA weather collections in the Iquitos station for the period 1999-2003 [37]. 

### Simulating *Medea* and KR in Skeeter Buster

Many variants of the *Medea* approach have been explored using general mathematical models [25,27,28]. For our purposes, we use a basic autosomal system with simple, feasible, genetic parameter values that are the focus of the current study. *Medea* and the linked anti-dengue gene are simulated as a single allele at a locus, where *M* denotes the *Medea*/anti-dengue allele and *m* denotes the wild-type allele. The *Medea* toxin is at least temporarily present in all embryos and could be associated with an additional fitness cost. We define the fitnesses of genotypes *mm*, *Mm*, *MM* as respectively 1, (1-*c*_M_) and (1-*c*_M_)^2^ where *c*_M_ is the value of the cost per construct. This cost is computed as an embryonic cost, i.e. an additional mortality factor applied to newly hatched eggs. Ward et al. [28] explore a wide variety of fitness costs to females, males, and embryos. In this study we consider that the lethality effect of the *Medea* element (the mortality of non-*Medea* offspring of a *Medea*-bearing mother) is 100%, i.e. none of the offspring survives. Although intermediate values of this factor are explored in other models [27,28] the empirical results of Chen et al. [25] indicate that 100% mortality can be achieved. In addition, we consider that the *M* allele can be associated with an additional, female specific fecundity cost *c*_F_ for both heterozygotes and homozygotes. This cost is expected to arise because of the *Medea*-specific embryonic effects, or because the associated anti-dengue gene is only expected to be expressed in females and is likely to exact a metabolic cost or have off target effects [13]. The simulations presented in the main text all assume *c*_F_=0.1.

 For the KR system, *K* and *R* are modeled as alleles on unlinked loci, with *K* and *R* denoting the killer and rescue transgenic alleles respectively, and *k* and *r* denoting their respective wild-type counterparts. We assume that the anti-dengue gene is completely linked to the *R* allele and thereby is modeled as a component of the *R* allele. Fitness costs associated with carrying *K* and/or *R* transgenes are handled similarly to the *Medea* case, with *c*_K_ and *c*_R_ as the respective embryonic fitness costs. No fecundity cost is modeled. Overall fitness of an individual is calculated multiplicatively across loci. Gould et al. [24] explore a number of scenarios for the phenotypic expression of the *K* and *R* alleles. Here, for simplicity, we assume that both *K* and *R* have dominant effects on killing and rescue, and that the lethality of the *K* allele and rescue by the *R* allele are complete. In other words, all individuals carrying at least one copy of the *K* allele are killed, provided they do not carry a copy of *R*. Individuals carrying at least one copy of *R* are insensitive to the lethality conferred by *K* alleles, whether one or two copies of *K* are present. 

### Release scenarios

 The main goal of this study is to examine how the spread of an anti-dengue gene will be affected by the specific ecology, movement behavior, and spatial/temporal heterogeneity in density and life stages of the resident and released mosquitoes. 

 The Skeeter Buster model has previously been used to examine the impacts of a variety of release strategies on the efficacy of a transgenic *Ae. aegypti* strain with a conditional female-killing gene [31] and the practical rationale for the different release strategies is detailed in that publication. In the current study we consider two possible life stages for release: adults or eggs. The release of adults is simply simulated by uniformly adding cohorts of male (or male and female) adults to all houses on the grid. To simulate the release of eggs, we introduce special containers with cohorts of male and female eggs (in equal numbers) into 10% of the houses. Since there are no practical means of separating male and female eggs, these releases result in both sexes emerging as adults. The released containers have a large initial supply of nutritional resources to guarantee favorable larval development, but they do not receive any additional food during the remainder of the simulation, are not available to female adults for oviposition, and are removed from the simulated area as soon as all released individuals have either died or emerged as adults. This ensures that no additional breeding sites are created in the simulated area. We designate 10% of houses in our simulated area as the release sites because a limited number of households are expected to participate. There is some mortality of eggs and immature stages. Therefore, we assess results of simulations with eggs in terms of the total number of adults expected to be produced over the entire release for each specific number of eggs released per release site. We also present results in terms of how many eggs or adults are released at single sites in a single week.

 We can therefore define a number of scenarios based on (1) the spatial pattern of release, (2) the life stage chosen for release, (3) the number of individuals released (and the number of separate releases) and (4) the parameters of the genetic strategy involved (namely the fitness cost values, i.e. *c*_*M*_ and *c*_*F*_ for *Medea*, *c*_*K*_ and *c*_*R*_ for KR). Because the model has many stochastic processes, 20 simulations were run for each scenario so that information on the variation in outcomes can be examined. Due to space limitations we only present detailed time series plots of allelic frequencies for a small number of simulations. For the most part, we present summaries of the status of the anti-dengue alleles three years after the releases. 

We selected the three-year time period as a compromise between two conflicting requirements. On the one hand, a shorter time frame would be favored by public health administrators and citizens of an affected community who would need to consent to the use of the transgenic approach. On the other hand, a sufficiently long evaluation time is required to properly assess the outcome of any particular release scenario. We observed (Figure S1) that shorter time frames would not be appropriate in that regard. A 1-year period (Figure S1A) does not allow *Medea* elements to significantly increase in frequency under all but the largest release scenarios, and would therefore lead to a significant overestimation of the required release size. A 2-year period (Figure S1B) would mostly correct this overestimation, but remains insufficient in most scenarios for the *Medea* elements to fully spread in the resident population. 

Because the ability of *Medea* elements to spread throughout populations is of primary interest, we categorize outcomes into classes based on the anti-dengue allele frequency after three years (‘full spread’: *freq*>0.8; ‘partial spread’: 0.2<freq<0.8; ‘no spread’: 0<freq<0.2; ‘lost’ freq=0). Note that ‘No spread’ indicates only the absence of spread to high frequencies within the timeframe considered in this study, and does not preclude the possibility of the element further increasing in frequency after a longer period. 

## Results

### Single release of homozygous *Medea* individuals

**Figure 1:**
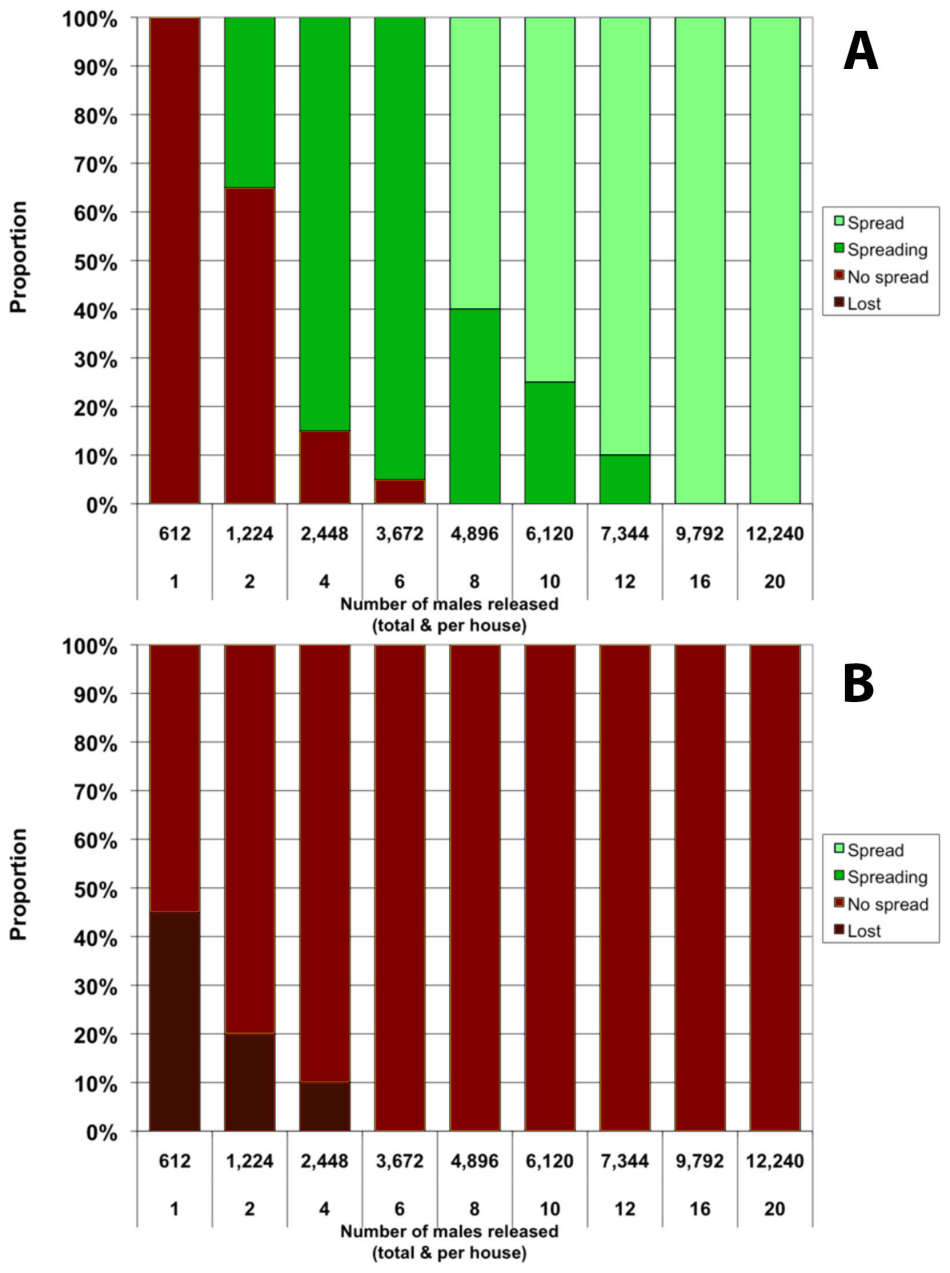
Single release of homozygous *Medea* adult males in every house. Proportion represents the fraction out of 20 simulations that reaches a given outcome 3 years after the first release. Outcomes are defined by the final allelic frequency *f* of the Medea construct in the population. ‘Spread’: *f*>0.8; ‘Spreading’: 0.2<f<0.8; ‘No spread’: 0<f<0.2; ‘Lost’: f=0. Both panels: *c*_F_=0.1. Top panel: *c*_M_=0; bottom panel: *c*_M_=0.1.

Because of the selfish spreading ability of *Medea* genetic constructs, a single uniform release of a sufficient number of transgenic adult males to each house can be enough to obtain high frequencies of the construct 3 years after release, even when a fecundity cost *c*_F_=0.1 is considered (Figure 1). If there is no cost *c*_*M*_ associated with the *Medea*/anti-dengue construct (Figure 1A), a single release of 16 males per house is sufficient to obtain final frequencies above 0.8 (full spread) in every replicated simulation. Note that with releases of 8 males per house, an increase in frequency of the construct is observed in every simulation; however, the allelic frequency of 0.8 is not always observed after 3 years. If the construct is associated with a fitness cost of *c*_M_=0.1 (Figure 1B), a single release of even 20 males per house is not sufficient for the transgenic element to begin spreading in the population. However, the *Medea* construct (and the linked anti-pathogen transgene) is not lost from the simulated populations if 6 or more males are released per house. Note that results are qualitatively similar when no fecundity cost *c*_F_ is considered (Figure S2), although in this case the construct is much less likely to be lost from the population in cases where it does not spread.

**Figure 2:**
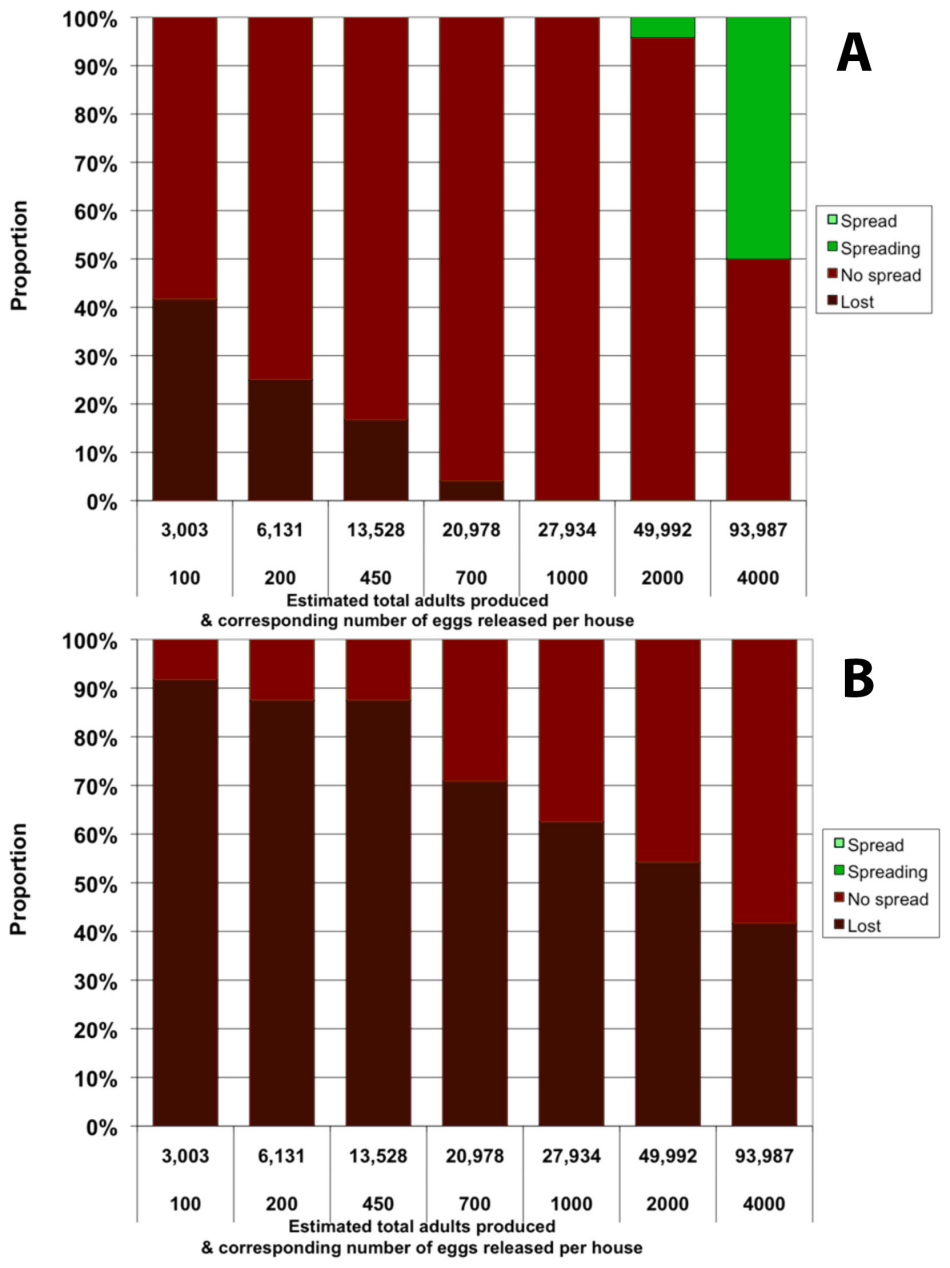
Single release of homozygous *Medea* eggs in 10% of houses. Proportion represents the fraction out of 20 simulations that reaches a given outcome 3 years after the first release. Outcomes are defined by the final allelic frequency *f* of the Medea construct in the population as in Figure 1. Both panels: *c*_F_=0.1. Top panel: *c*_M_=0; bottom panel: *c*_M_=0.1. The total number of adults produced from released eggs is estimated based on the average adult production of an egg cohort of corresponding size.

When *Medea*-bearing eggs are released in selected sites into the population, the *Medea* element attains partial spread in some simulations with a single release of 2000 eggs per site or more (Figure 2A). However, this method is quantitatively much less efficient than the uniform release of adult males (comparing the equivalent total number of adult males needed by each method to attain the same degree of spread). When eggs are the release stage, the *Medea* construct does not spread into the population in any of our simulations if it is associated with a fitness cost (Figure 2B). The release into only 10% of houses is problematic because it requires the *Medea* construct to spread spatially as well as temporally. Here again, results are qualitatively similar whether *c*_F_=0.1 (Figure 2) or *c*_F_=0 (Figure S3). The main difference is that, in the presence of a dominant fecundity cost *c*_F_, the construct is more likely to be lost from the population when it is released at low frequencies that do not permit spread. This is likely to be a desirable trait for the strategies examined here, as it may help prevent unwanted spread to non-target populations. 

### Multiple releases of homozygous *Medea* individuals

**Figure 3:**
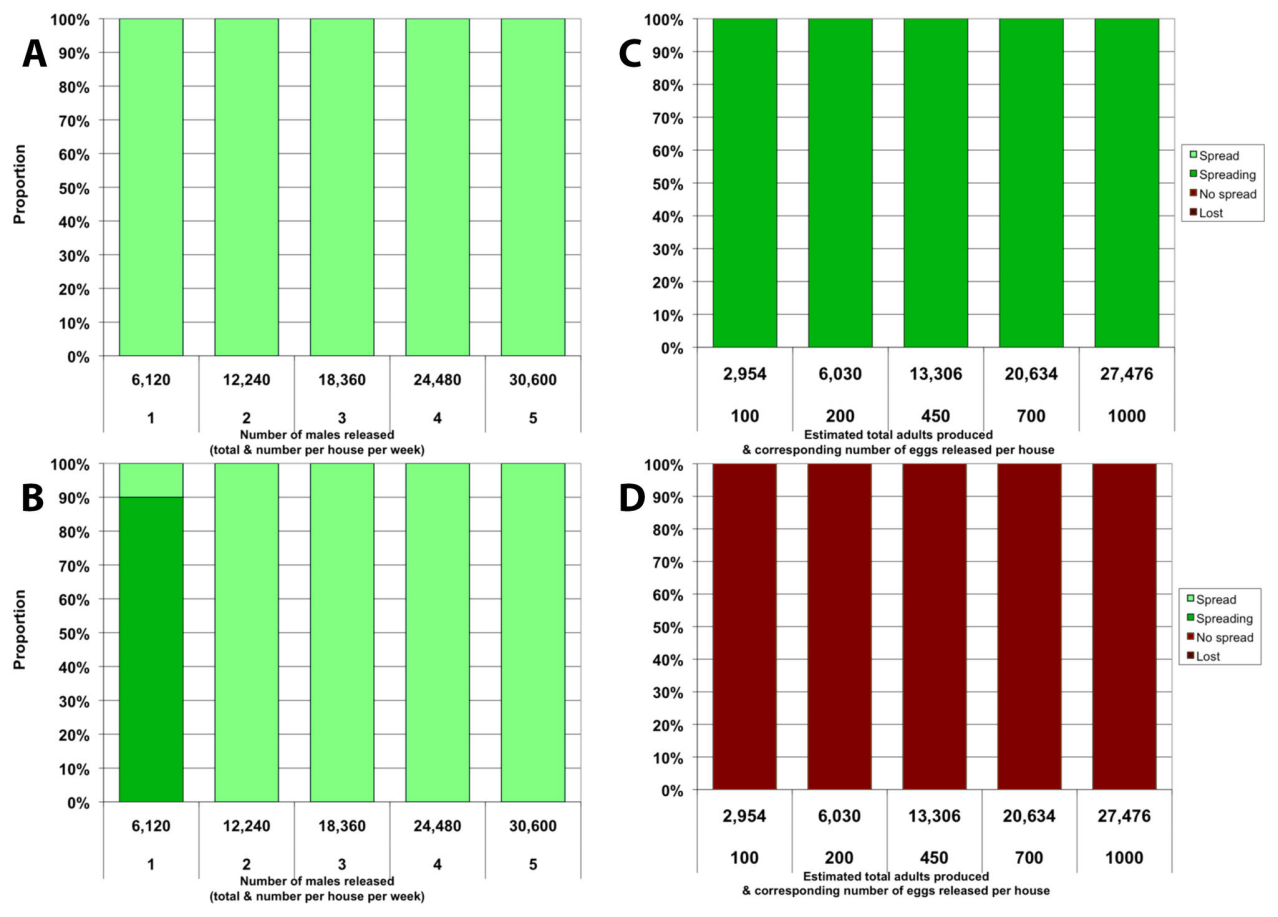
Multiple releases of homozygous *Medea* adults and eggs. Proportion represents the fraction out of 20 simulations that reaches a given outcome 3 years after the first release. Outcomes are defined by the final allelic frequency *f* of the Medea construct in the population as in Figure 1. All scenarios involve 10 weekly releases. All panels: *c*_F_=0.1. Top panels: *c*_M_=0; bottom panels: *c*_M_=0.1. Left column: release of adults in every house. Right column: release of eggs in 10% of houses.

When 10 consecutive weekly releases of homozygous *Medea* adult males are simulated, the spread of the construct is more efficient (i.e. fewer total males released for the same outcome) than in the single releases (Figure 3). If there is no embryonic fitness cost, high final frequencies are achieved with releases as low as 1 male per house per week (Figure 3A). This type of release represents a total of 6,120 males for the whole release program, a number that does not yield consistent full spread within 3 years when all of the transgenic males are released in a single event (Figure 1A). The difference is more pronounced when the construct is associated with an embryonic fitness cost (Figure 3B). In that case, 10 releases as low as 2 males/house/week, corresponding to a total of 12,240 males, result in consistent full spread within 3 years, whereas the same number in a single release does not result in any spread of the construct (Figure 1B). 

 When eggs are released, multiple releases are also more favorable for the spread of the transgene if it has no embryonic fitness cost (Figure 3C). Partial spread is observed for releases as low as 100 eggs per house per week, whereas spread was not observed at all with single releases of that same total number of eggs (Figure 2A). If there is a fitness cost (Figure 3D), spread is not observed any more than with a single release, although multiple releases still appear somewhat beneficial in that the linked anti-dengue transgene is never lost from the 20 simulated populations (contrary to the single release case, Figure 2B). 

### Single release of homozygous *Medea* adults of both sexes

**Figure 4:**
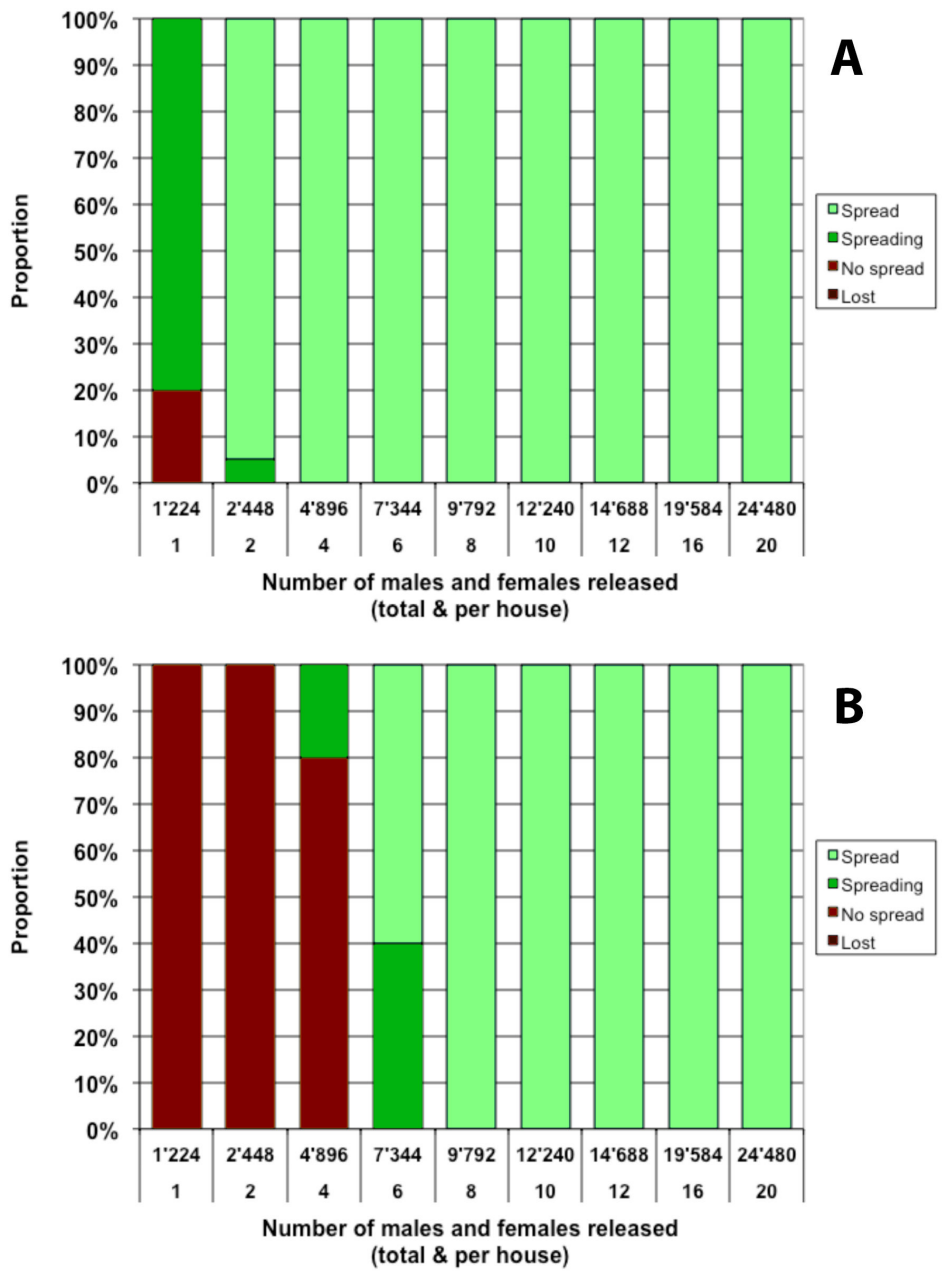
Single release of homozygous *Medea* adult males and females in every house. Proportion represents the fraction out of 20 simulations that reaches a given outcome 3 years after the first release. Outcomes are defined by the final allelic frequency *f* of the Medea construct in the population. Categories defined as in Figure 1. Both panels: *c*_F_=0.1. Top panel: *c*_M_=0; bottom panel: *c*_M_=0.1.

When releasing adults into the resident population, it is generally considered preferable to release only males, since they do not bite and*, a fortiori*, do not transmit disease. Unfortunately, this imposes a strong limitation on the frequency that a newly introduced genetic element can reach one generation after release. Even under the most favorable assumptions (all females in the resident population are virgin and all mate with released males) the frequency of the transgene cannot exceed 0.50 in the next generation. To overcome this limitation it is necessary to release homozygous transgenic females along with the males. We show that the combined release of males and females substantially improves the fate of a *Medea* allele in our simulations. When adults are released once in every premise in the simulated area, full spread of the *Medea* allele is observed in more than 90% of cases when releasing as little as 2 males and 2 females per house (Figure 4A), whereas a male-only release required at least 12 males per house to achieve the same result. The difference is even more striking when the transgene is associated with a cost: whereas male-only releases failed to achieve even partial spread with up to 20 males per house, full spread is observed consistently in our simulations with a single release of 8 males and 8 females per house (Figure 4B).

### Single release of homozygous KR individuals

**Figure 5:**
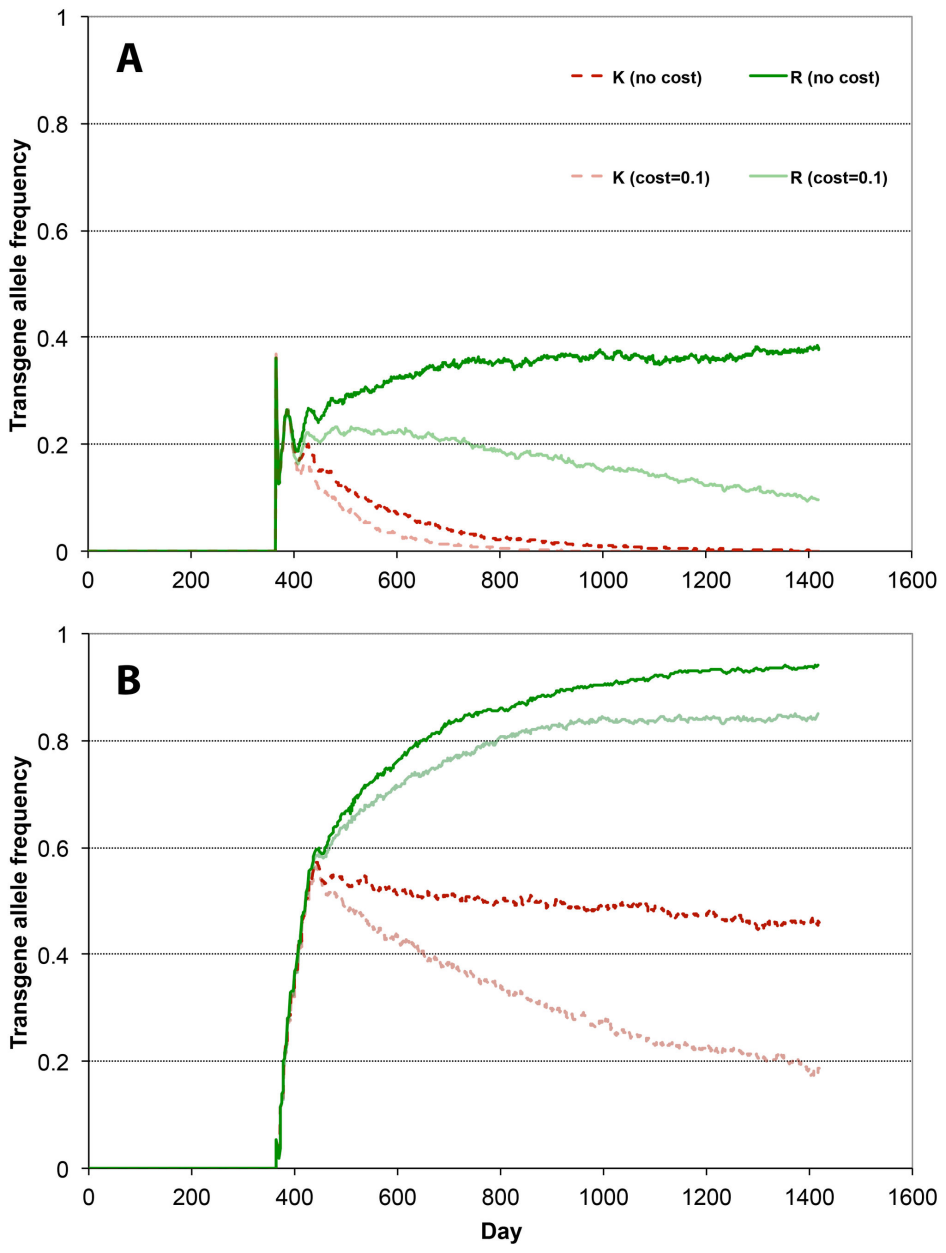
Time series of allele frequencies with releases of homozygous KR adult males in every house. The trajectories represent the change in frequencies of K allele (red, dashed lines) and R allele (green, solid lines) in the population in one simulation of releases of KR males in every house. The model is run for one year without release to establish the resident population, releases then start on day 365. Dark lines: *c*_K_ = *c*_R_ = 0. Light lines: *c*_K_ = *c*_R_ = 0.1. Top: single release of 100 males per house. Bottom: 10 weekly releases of 10 males per house each. In both cases, the total number of males released is 61,200.

**Figure 6:**
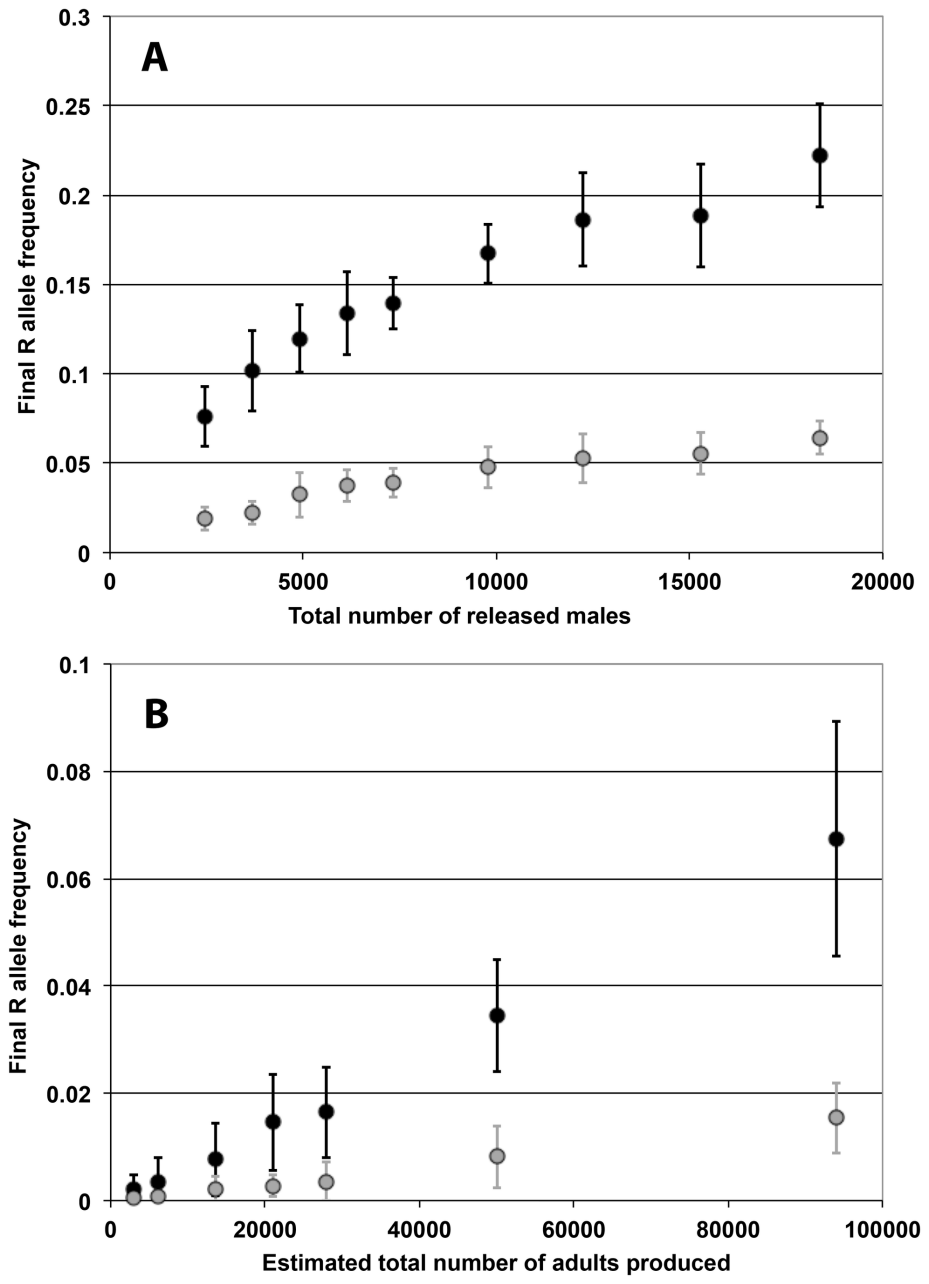
Final R allele frequency with single release of homozygous KR individuals. For each scenario the final frequency of the R allele in the population is plotted (20 replicates, average ± SD). Top: single release of adult males in every house. Bottom: single release of eggs in 10% of houses. Dark: *c*_K_ = *c*_R_ = 0. Light: *c*_K_ = *c*_R_ = 0.1. Note that the Y-axis is different in Figure 6 A and B.

A single uniform release of homozygous KR male adults, with numbers as high as 100 males per house (61,200 total males), results only in partial spread of the R allele in the population, with a final frequency remaining below 0.40 (Figure 5A). The final frequency of the R allele increases with the number of males released per house (Figure 6A), but remains at low values. If there is a fitness cost associated with carrying the R allele, the frequency of R does not reach values as high as in the no-cost case, and starts to decrease once the K allele has become rare in the population (Figure 5A). Consequently, with such a fitness cost, the final frequency of the R allele is much lower (Figure 6A). 

 As in the *Medea* case, we also consider the case of a release of eggs in 10% of the houses. Similarly to our prior observations, this type of release appears less efficient in the case of a single release (Figure 6B). The final frequency of the R allele does increase with the total number of eggs released. However, at an equal number of adult males produced, the frequency that is reached by releasing eggs is considerably lower than that obtained by releasing adults, whether transgenes are associated with a cost or not. 

### Multiple releases of homozygous KR individuals

With 10 consecutive weekly releases of KR individuals, the final frequencies of R that can be observed are notably higher. For example, in one simulation with 10 releases of 10 males per week in every house (total number of 61,200 males), the R allele reaches a frequency over 0.90 after three years when there is no cost. When the R allele frequency is above 0.90, the population has less than 1% rr individuals that can transmit disease (see Gould et al. [24]). When there is a fitness cost (Figure 5B) the R allele frequency is still over 0.80 (less than 4% rr individuals). In contrast, with a single release of the same number of adult males, the final R frequencies are below 0.40 and 0.10 respectively (Figure 5A). In each of the simulations in Figure 5, the frequency of the K allele continues to decline throughout the period after release, and as expected, the rate of decline is correlated with the frequency of the rr individuals in the population [24]. The R allele frequency never declines if there is no fitness cost associated with it. In the case with a fitness cost to the R allele and 10 releases (Figure 5B), the R allele does not decline during the 3-year period because the K allele is at a high enough frequency to balance the effect of the fitness cost. 

**Figure 7:**
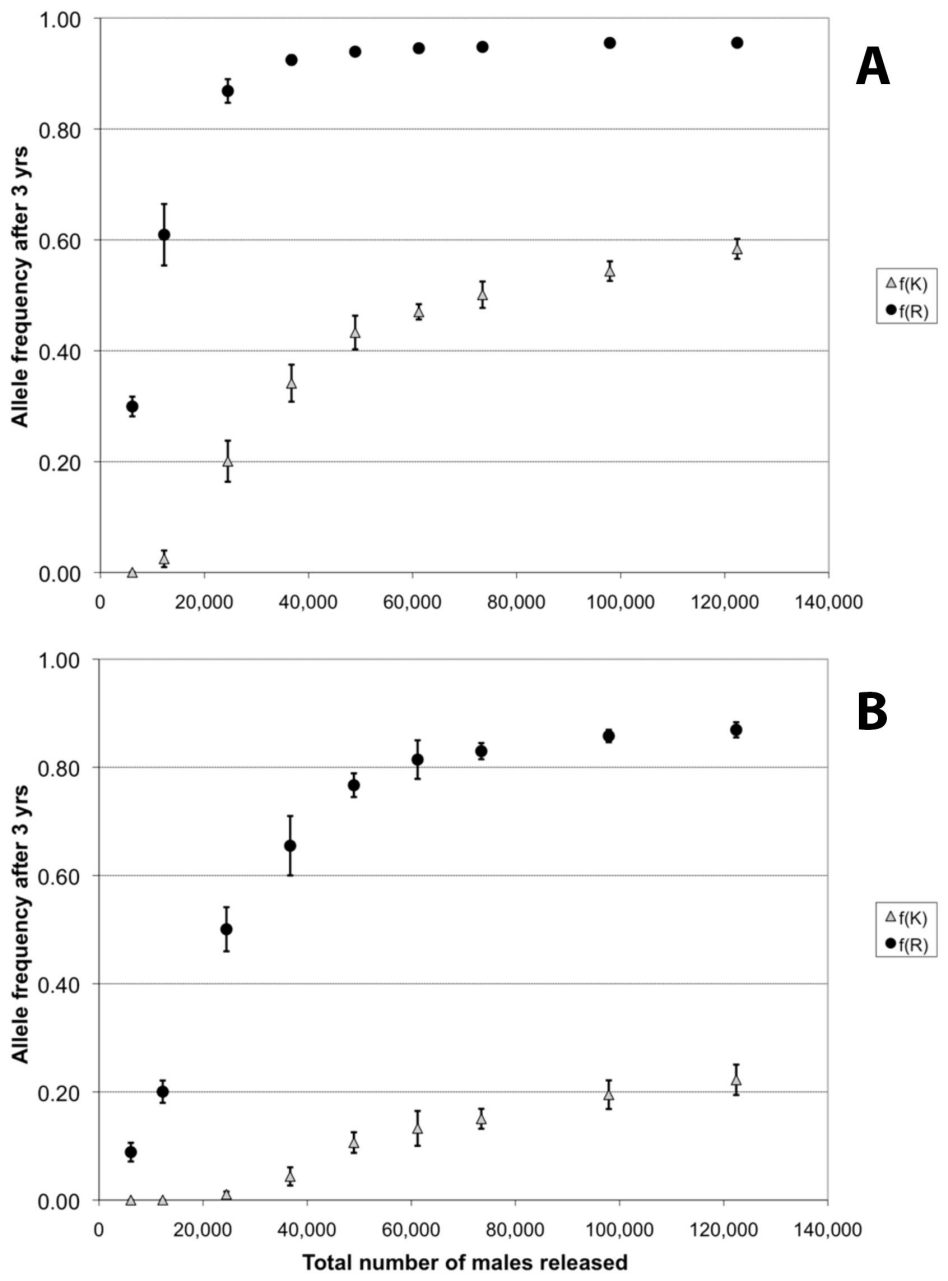
Final R and K alleles frequencies with multiple releases of homozygous KR adult males in every house. For each scenario the final frequency of each allele in the population after three years is plotted (20 replicates, average ± SD). Top: *c*_K_ = *c*_R_ = 0. Bottom: *c*_K_ = *c*_R_ = 0.1. Dark circles: final frequency of R allele. Light triangles: final frequency of K allele.

Figure 7A summarizes results of 20 simulations for each of a set of release parameters and shows that an R-bearing construct with no cost can consistently reach frequencies of approximately 0.95 with a total number of released males as low as 40,000 (Figure 7A) provided these releases are conducted over a 10 week period. The final frequencies of the R allele are lower if that construct is associated with a cost (Figure 7B), but frequencies above 0.80 can still be reached and maintained for three years with releases of 60,000 males or higher. This results in less than 4% of the females being capable of dengue virus transmission.

## Discussion

 There have been a number of mathematical models of the dynamics of *Medea* [25,27,28] and one of the KR system [24]. All of these are deterministic models that provide useful general insights about the dynamics of these gene drive systems, but they lack the detail needed to assess their practicality in suppressing a vector-borne pathogen in a heterogeneous urban environment within a reasonable period of time.

 The current study helps to fill that gap by use of a detailed spatial model to explore how a variety of operational factors in a release program could affect the spread of anti-dengue genes in a specific city. In the case of *Medea*, it is clear that releasing males over a period of time is more efficient than single releases in terms of the total number of mosquitoes that must be released to achieve the equivalent frequency of the anti-dengue genes three-years after initiating releases. Release of adult males and females is more efficient than release of only males, at least in part because this enables some mating between transgenic insects in the release generation, and this boosts the starting anti-dengue allele frequency. 

 Release of male and female adults over time is the most efficient approach of those explored here. Even though egg releases result in transgenic male and female adults mating, this approach is less efficient than male only releases. The low efficiency is at least in part due to the fact that eggs must be raised within a limited number of households and, therefore, the anti-dengue genes need to spread spatially as well as over time. It is likely that with the high number of eggs released in single households, there will be larval competition for food, and this will negate part of the boost in frequency that could accompany the mating of transgenic males and females. 

 From a societal perspective, it is thought that the sole release of males would be more acceptable even though all of the released mosquitoes are expected to carry anti-dengue genes. This may remain the case with transgenic mosquito releases, but recently large numbers of female mosquitoes carrying *Wolbachia* were released in Australia without public opposition because of data indicating that these females would not transmit dengue [38].

 In the case of the KR system, our model predicts that release over time would be the most efficient approach and that release of eggs in 10% of the houses is much less effective than release of adult males. Results of Gould et al. [24] indicated that whenever there was a fitness cost to the construct that included the rescue allele and the anti-dengue allele, the construct would eventually be lost from the population. What the simple model used by Gould et al. could not accurately predict was what the time frame would be for the increase and decrease in the frequency of this construct in a resident population under field conditions. The current simulations demonstrate that the frequency of the anti-dengue allele could increase above 0.90 in three years with modest levels of mosquito releases. The outputs also demonstrate that the construct with the K allele would begin to decline immediately after releases ended, even when there was no fitness cost associated with the K allele (when in the presence of an R allele). In the time series plots, it is clear that once the K allele frequency declines toward extinction, the R allele begins to decline if there is a fitness cost associated with it. This all suggests that the KR gene drive system could be used for spatially and temporally limited testing of the impact of an anti-dengue gene.

 In the current study, we evaluated efficiency based on the total number of transgenic individuals that had to be released in a specific operational approach to achieve a given level of spread of the anti-dengue gene in the population. We made no attempt to estimate the cost of production and release of the transgenic insects. It is expected that the cost of release per egg would be different than the cost of release per adult. Furthermore, when only males are released a factory would still need to rear the females until pupation in order to separate the sexes. Some of the females could be used for breeding but many would be discarded. 

 We fully realize that in real world applications there would be no way to release the same number of adults in every house in a city or to uniformly select every tenth house for egg releases. We examined these extreme spatial release scenarios to gain a qualitative sense of the factors impacting efficacy. If and when the development of transgenic strains advances to the point at which large-scale field releases are being planned, the detailed model could be used in coordination with public health authorities to conduct simulations of the specific types of releases that are being considered. Of course, the Skeeter Buster model, as used in the current study was parameterized for Iquitos, Peru. Re-parameterization would be needed to fit other locations. 

 Even with the limitations outlined above, our exploration of the dynamics of *Medea* and KR in *Ae. aegypti* populations with the Skeeter Buster model offers the only detailed assessment of how tactical and operational factors could impact the efficiency of a disease suppression program based on gene drive. Given the large investments that are being made in molecular genetics of gene drive systems for this purpose, there is a need for a better understanding of how they will behave under field conditions. Our study lays out an example of how models like Skeeter Buster could be developed and used to guide the evaluation of other mechanisms and other vector systems.

## Supporting Information

Figure S1 **Outcome of single release of homozygous *Medea* adult males in every house evaluated 1 year (**A**), 2 years (**B**) or 3 years (**C**) after release.** Proportion represents the fraction out of 20 simulations that reaches a given outcome after the corresponding time period. Outcomes are defined as in Figure 1. Parameters as in Figure 1A: *c*_F_=0.1, *c*_M_=0.
(TIF)

Figure S2 **Single release of homozygous *Medea* adult males in every house (no fecundity cost).** Proportion represents the fraction out of 20 simulations that reaches a given outcome 3 years after the first release. Outcomes are defined by the final allelic frequency *f* of the *Medea* construct in the population as in Figure 1 Both panels: *c*_F_=0. Top panel: *c*_M_=0; bottom panel: *c*_M_=0.1.
(TIF)

Figure S3 **Single release of homozygous *Medea* eggs in 10% of houses (no fecundity cost).** Proportion represents the fraction out of 20 simulations that reaches a given outcome 3 years after the first release. Outcomes are defined by the final allelic frequency *f* of the *Medea* construct in the population as in Figure 1. Both panels: *c*_F_=0. Top panel: *c*_M_=0; bottom panel: *c*_M_=0.1. The total number of adults produced from released eggs is estimated based on the average adult production of an egg cohort of corresponding size.
(TIF)
