# Supplementary figures and images for: Modeling the Dynamics of a Non-Limited and a Self-Limited Gene Drive System in Structured Aedes aegypti Populations
Source: PLoS One. 2013 Dec 10;8(12):e83354. doi: 10.1371/journal.pone.0083354 (PMC3858347; doi:10.1371/journal.pone.0083354)

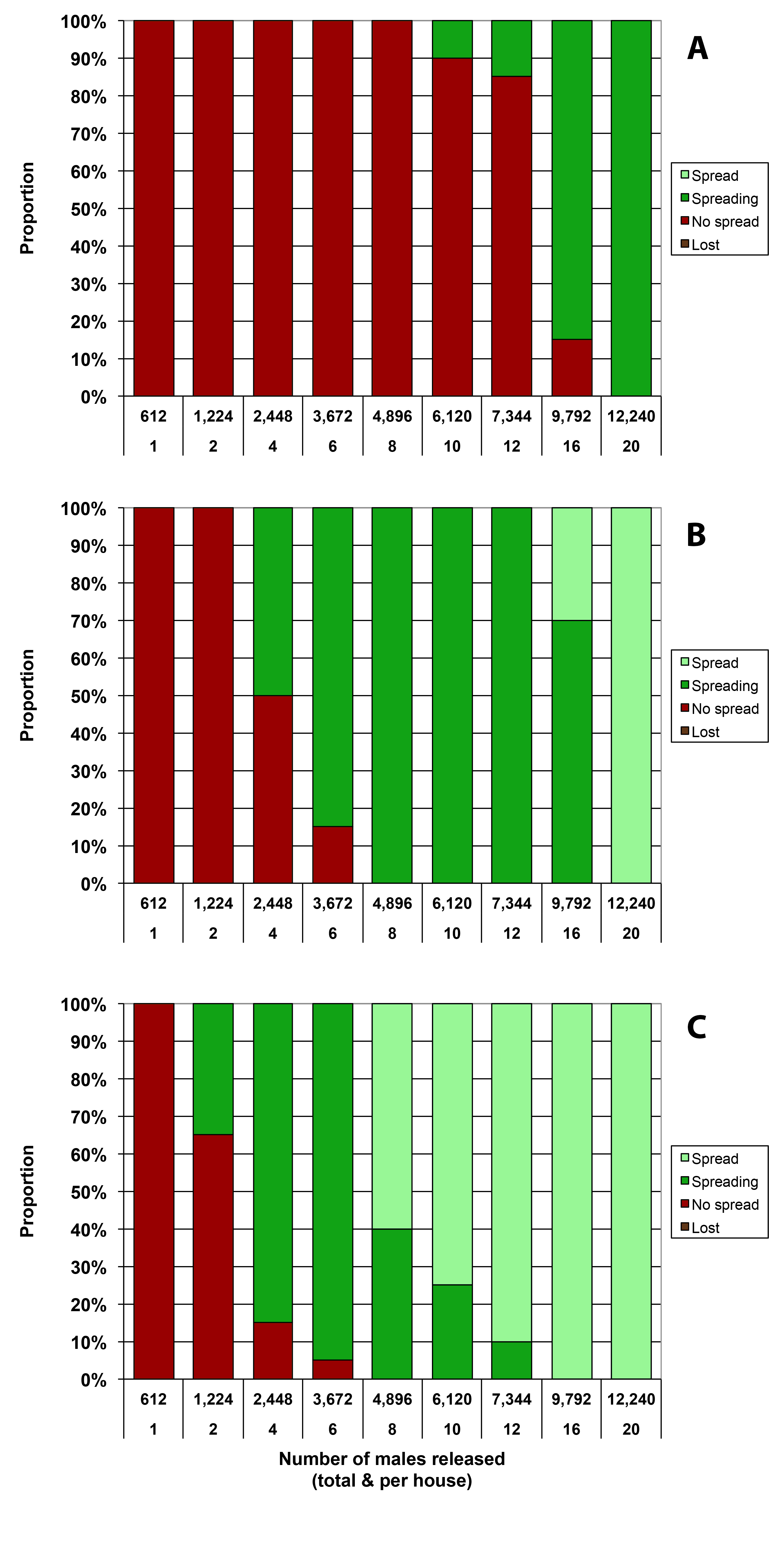

Supplement: Figure S1 — Outcome of single release of homozygous Medea adult males in every house evaluated 1 year (A), 2 years (B) or 3 years (C) after release. Proportion represents the fraction out of 20 simulations that reaches a given outcome after the corresponding time period. Outcomes are defined as in Figure 1. Parameters as in Figure 1A: c F=0.1, c M=0. (TIF) [file pone.0083354.s001.tif]

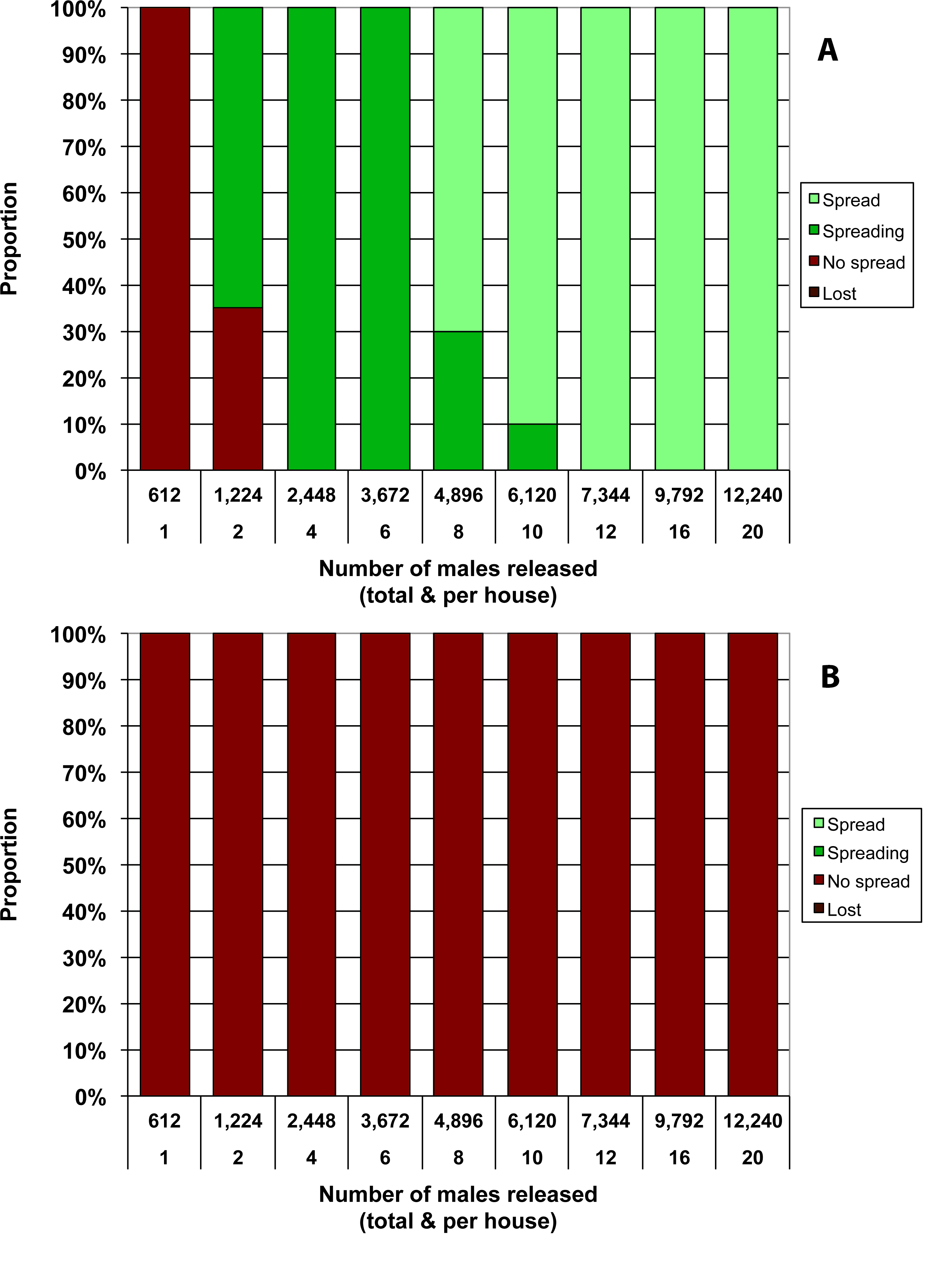

Supplement: Figure S2 — Single release of homozygous Medea adult males in every house (no fecundity cost). Proportion represents the fraction out of 20 simulations that reaches a given outcome 3 years after the first release. Outcomes are defined by the final allelic frequency f of the Medea construct in the population as in Figure 1 Both panels: c F=0. Top panel: c M=0; bottom panel: c M=0.1. (TIF) [file pone.0083354.s002.tif]

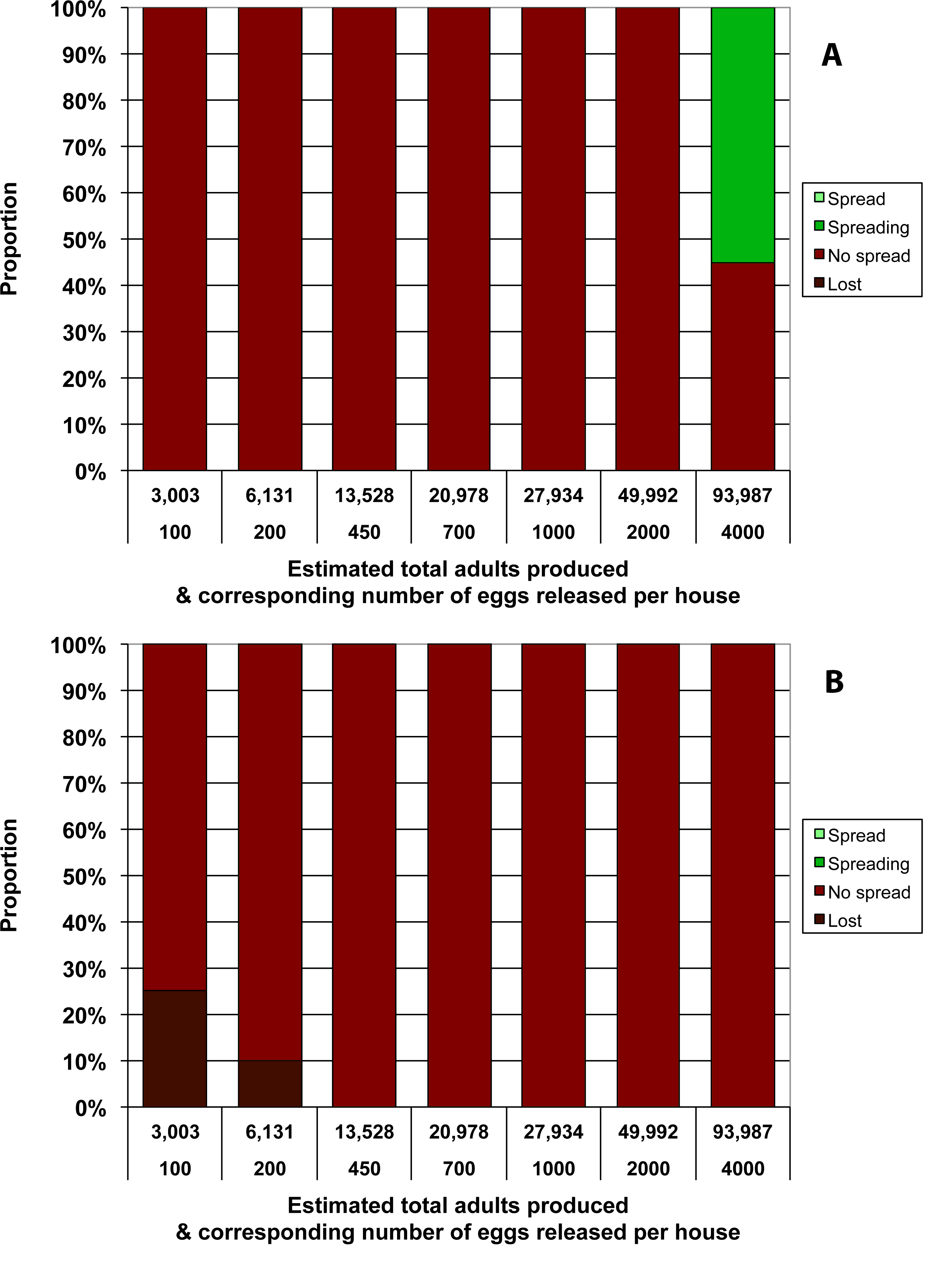

Supplement: Figure S3 — Single release of homozygous Medea eggs in 10% of houses (no fecundity cost). Proportion represents the fraction out of 20 simulations that reaches a given outcome 3 years after the first release. Outcomes are defined by the final allelic frequency f of the Medea construct in the population as in Figure 1. Both panels: c F=0. Top panel: c M=0; bottom panel: c M=0.1. The total number of adults produced from released eggs is estimated based on the average adult production of an egg cohort of corresponding size. (TIF) [file pone.0083354.s003.tif]
